# Temporal dynamics of justice climate and team innovation

**DOI:** 10.3389/fpsyg.2022.699319

**Published:** 2023-02-01

**Authors:** Neha Tripathi, Sukanya Sangar

**Affiliations:** Human Resources Management, Indian Institute of Management Ahmedabad, Ahmedabad, India

**Keywords:** justice climate, group emotions, social contagion, positive affect, negative affect, team innovation

## Abstract

Team innovation—exploration and exploitation of useful and novel ideas by a team has been a topic of great importance for organizations in today’s dynamic, complex, and competitive environment. Grounded in the social contagion theory of justice, we theorize a justice-to-innovation processual model based on within-team justice climate occurrences that change over time. We posit that collective and shared justice perceptions of team members construct dynamically based on justice-related work events. Within teams, state justice climate level and strength (represented by the Mean and the low-SD scores of individual team members in the moment or an episode) are important precursors of team innovation. The proposed theoretical model explicates an emotional contagion process arguing that positive and negative team affect states mediate the relationship between state justice climate and team innovation. Positive/negative team affect states result in collective actions and team interactions that foster/hinder team innovation. The present article significantly contributes to the development of the dynamical models of justice and innovation for teams where most research is confined to static models of justice climate.

## Introduction

In the past three decades, team^1^ innovation—generation and implementation of useful ideas by a work group or team (Amabile, 1988; West and Farr, 1990) has been studied in great depth, identifying a multitude of team-level variables that foster or hinder team innovation (Hülsheger et al., 2009). Although innovation is conceptualized as a dynamic process (process-oriented models by Van de Ven et al., 1989; King, 1992), the current state of the science on team innovation is critiqued for lack of studies that focused attention on investigating temporal and dynamic processes underlying team innovation (Anderson et al., 2014). It is noteworthy that, at the individual level, scholars have shown empirical evidence of the transient nature of an individual’s creativity (experience-sampling study, Bledow et al., 2013) and innovative behaviors (daily diary study, Zacher and Wilden, 2014). However, at the team level, the research on dynamic models of team innovation is still in its infancy. Elucidating the need for studies focusing on dynamic processes underlying team innovation, Anderson et al. (2014) called for future research to “adopt a ‘*momentum perspective*’ to examine the effects of changes in key variables over time and how these impinge upon subsequent innovativeness.”

Studying the dynamic relationship between a team’s shared justice perceptions and innovation is important for two reasons. First, since innovation involves competing demands of exploration and exploitation (Kanter, 1988; March, 1991; Rosing et al., 2011); innovation entails greater uncertainty and ambiguity for the parties involved. People rely on their justice perceptions in times of uncertainty and change (Van den Bos, 2001; Dayan and Colak, 2008; Akgün et al., 2010). Justice becomes a pivotal antecedent element to foster team innovation. Second, a few empirical studies, mostly cross-sectional, show that justice climate influences team creativity and team learning, the key factors associated with team innovation (Dayan and Colak, 2008; Akgün et al., 2010). These studies serve as an important starting point; however, they lack explicating of the process of *how* justice affects innovation at the collective level. For instance, justice-related events occur on a daily basis, but how do these events transform the justice climate across time to influence collective and shared team emotions, interactions, and innovation-related activities? An integrative theory of justice and innovation should employ a sociopsychological lens to illuminate within-team changes in collective justice perceptions—varying occurrences of team climate across time—to affect team innovation.

Social contagion theory for justice (Degoey, 2000, p. 51) contends that the “ambiguous and emotionally charged nature of justice-related events compel organizational actors to engage in social ‘talk’ and arrive at a shared, socially constructed interpretation of justice,” therefore, shared justice perception is important in mobilizing collective actions. Thus far, justice climate is assumed to be a static construct that is built over a long period and then remains stable over time. However, such an assumption has been critiqued arguing dynamism in justice climate that the *level* (the Mean) and *strength* (low SD) of the team’s justice climate is transient and may change within a short time because people encounter discrete justice events daily in the workplace (Rupp and Paddock, 2010; Jones and Skarlicki, 2013) and continually engage in socializing, social information sharing, and collective sense-making with other team members.

The present article elaborates on the emotional contagion process (Degoey, 2000) that leverages the effect of state justice climate on team innovation from the team members’ collective affective response, that is team’s shared positive and negative affectivity. Similar to any emotion, mood or affect state drives some actions or behavioral responses among individuals. We argue that emotions are shared in a team and propel interactions or collective actions among team members, which in turn influence team innovation. In a positive team affect state, team members share positive feelings in the moment/episode. Positive affect enables cognitive and social spontaneity (Broaden-and-build; Fredrickson, 2001), which may leverage group interactions by communicating, affirming, and building on one another’s ideas (Rhee, 2007). Similarly, a negative team affect state represents the team’s shared negative feelings of team members in the moment/episode. Negative affect triggers either desirable or counterproductive actions depending on attribution about in/justice events (Degoey, 2000; Folger and Cropanzano, 2001; Barclay et al., 2005). Negative team affect states may either leverage outcome-focused interactions (planning, monitoring, and critical evaluation, Rhee, 2007) or counterproductive interactions (silence, collective criticism, and group-based antisocial behaviors, Degoey, 2000), depending on whether injustice is attributed internally or externally.

**FIGURE 1:**
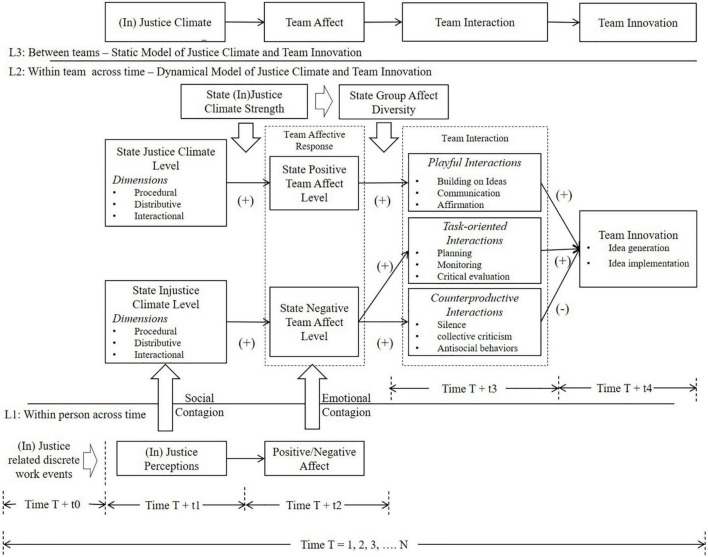
Conceptual model.

Not only team affect *level* but also team affect *diversity*—high variability in affective states experienced by team members (Barsade and Knight, 2015) influences team members’ interactions. Tiedens et al. (2004, p. 164) argue that “groups do not transform members into clones. Variance persists in even the most homogeneous groups … every group is composed of people who feel and express distinct emotions.” Affect diversity can arise in teams and play an important role in how groups appraise and act upon the (in) justice events collectively, either *polarizing* or *mitigating (normalizing)* the collective affective experience, influencing team members’ interactions. Overall, based on the social contagion theory of justice (Degoey, 2000), the present article provides a theoretical framework to explain the emotional contagion process of justice events to form transient and momentary states of justice climate to influence team innovation across time. Shared positive/negative affective states of team members mediate the effect of state (in) justice climate on team innovation. Positive and negative team affect (both the Mean and the SD) further influence team members’ interactions (Conceptual model, Figure 1).

By explicating a dynamic process of team innovation *via* the emotional contagion of justice within a team across time, the present theoretical framework contributes to the literature in two important ways. First, an event-based perspective to theorize team level processes changing across time is underdeveloped both in theoretical insights and in empirical evidence. The extant theories have predominantly focused on the static nature of team climate, assuming that the team climate “stabilizes” over time. Scholars now caution against this underlying assumption and critique that this has, in the majority, resulted in cross-sectional studies (Hülsheger et al., 2009). Little is known about how dynamic and changing team climate influences team innovation over time (Anderson et al., 2014). Presenting a dynamic processual model for team innovation significantly contributes to the advancement of theories of team innovation by integrating a *momentum perspective* illuminating on temporal effects of a justice climate on team innovation.

Second, a widely accepted framework for team innovation is the input–process–output model (Ilgen et al., 2005), arguing that the team input variables—the team composition and structure fuel team-level processes, and facilitate team innovation (refer to meta-analysis, Hülsheger et al., 2009). Such structural antecedents of team innovation are investigated in greater depth. However, scholars point toward motivation as an especially critical driver of team innovation, where the team members collectively direct effort to generate and implement new processes, procedures, or products (Klein and Sorra, 1996; Baer, 2012). The team-level inputs (e.g., leadership, group norms, shared goals, work design, and feedback, Chen and Kanfer, 2006) influence the team’s motivation for innovation. At the individual level, organizational justice is known to be a strong motivator for creativity (Janssen, 2004) and innovation (Ng et al., 2010; Simmons, 2011). At the collective level, the social and emotional contagion of justice must serve as a salient motivation to foster team innovation. The present article advances theories of team innovation by illuminating collective justice as a key motivational antecedent to team innovation.

## Theory and propositions

### Justice climate and team innovation

Justice climate refers to the group-level understanding of fairness in how a workgroup is treated (Naumann and Bennett, 2000; Colquitt et al., 2002). The present article conceptualizes team justice climate as a construct that changes over time as team members encounter justice-related work events during their routine work (Rupp and Paddock, 2010; Jones and Skarlicki, 2013) and while they socialize and share information with other team members (Li and Cropanzano, 2009). The Justice climate of a team consists of two components: the climate level—the favorability of the unit mean aggregated over all team members, and the climate strength—the congruence of unit members’ justice perceptions of team members (Colquitt et al., 2002). Both justice climate level and strength are dynamically constructed and change over time. As a state, justice climate level is defined as “*in-the-moment or localized (episodic) shared justice perceptions of the team members on how fairly the team is being treated.*” Similarly, state justice climate strength is defined as *low dispersion in “in-the-moment or localized (episodic) justice perceptions among team members on how fairly the team is being treated.”*

Innovation, fundamentally, refers to the “successful introduction into an applied situation of means and ends that are new to the situation” (Mohr, 1969, p. 112), which typically requires the generation of new ideas, technologies, or processes followed by realization or implementation of these ideas/approaches in the organizational settings (Amabile, 1988; Kanter, 1988). Accordingly, innovation encompasses two components: (a) idea creation or generation, and (b) idea implementation (West and Farr, 1990; De Jong and Den Hartog, 2010). Organizational innovation is possible only through team-level efforts. Anderson and West (1998) noted that “it is often the case that an innovation is originated and subsequently developed by a team into routinized practice within organizations.” Team innovation requires both exploration and exploitation activities (March, 1991). While exploration involves creativity, experimentation, and risk-taking; exploitation includes implementation, effectiveness, and goal attainment (March, 1991). Teams engage in exploration and exploitation activities dynamically (punctuated equilibrium, Gersick, 1991; Romanelli and Tushman, 1994, and ambidexterity, Andriopoulos and Lewis, 2009; Bledow et al., 2009). The predictors for team innovation include structural antecedents (e.g., team size and longevity), and psychological antecedents (cohesiveness and participative safety) (Anderson and King, 1993; Hülsheger et al., 2009). Scholars have pointed out links between team justice climate and team innovation during new product development (team creativity, team learning, Dayan and Colak, 2008; Akgün et al., 2010). A perceived justice climate played an important role in the successful deployment of new policies and procedures during organizational change (Caldwell et al., 2004).

Shared justice perception in a group is formed through social contagion (Degoey, 2000). The social contagion process is facilitated in several ways, including social comparison (Festinger, 1954), social information processing (Salancik and Pfeffer, 1978), and collective sense-making (Weick, 1995). In social comparison, individuals base their fairness judgments about a situation on social cues and information from others that can help them in interpreting the situation accurately (Festinger, 1954). Within teams, members converse on the work events with other members to better understand the situation. The team members rely on one another’s points of view in interpreting what justice aspects are worth evaluating and value others’ interpretations of justice-related events. Social information or social cues such as the opinions of co-workers, influence team members in assessing perceived fairness (Jones and Skarlicki, 2005; Hollensbe et al., 2008). The team members engage in collective sense-making to interpret the intended meaning of (in) justice-related events (Rupp and Paddock, 2010; Jones and Skarlicki, 2013). When members encounter a situation that is ambiguous or unexpected, they attempt to make sense of that situation, often through using social cues and information from others in the group (Weick, 1995; Roberson, 2006).

Scholars define three dimensions of justice climate—distributive, procedural, and interactional justice climate. Distributive justice climate refers to shared perceptions of fairness of reward and resource distribution, or outcome fairness (Equity theory, Adams, 1965; Leventhal, 1980). Procedural justice climate refers to shared perceptions of fairness in organizational decision-making processes and procedures (Relational theory, Thibaut and Walker, 1975; Lind and Tyler, 1988; Tyler and Blader, 2000). Interactional justice is comprised of interpersonal and informational justice. Interpersonal justice climate refers to shared fairness perceptions that one is treated with dignity and respect (Bies and Moag, 1986) and informational justice climate refers to shared fairness perceptions of the availability of truthful and adequate explanations for decisions (Greenberg, 1993). In the following, we elaborate on how the dynamic nature of distributive, procedural, and interactional justice climates foster team innovation.

#### State distributive justice climate level and team innovation

State distributive justice climate level refers to a team’s in-the-moment or localized (episodic) shared perceptions (the mean) for fairness of outcomes—the allocation of rewards and resources. Individuals perceive distributive fairness as they evaluate balance or equality in the inputs and the outcomes (Adams, 1965). In the context of a team, team members are motivated to seek out peers’ opinions about how they view the fairness of the outcomes. As individuals encounter outcome-related justice events such as the allocation of rewards and resources, they seek out their peers’ fairness perceptions (Folger et al., 1979), and engage in a group discussion by covert or overt means. Furthermore, people show higher confidence when fairness perceptions are formed collectively.

A team’s in-the-moment or localized (episodic) shared perceptions of outcome fairness influence team innovation for two reasons. First, distributive justice facilitates higher trust in management (Dayan et al., 2009). With higher trust in the management, the team members are likely to value and embrace the organizational vision and goals for innovation (West, 1990; West and Anderson, 1996) and mobilize their effort collectively to attain the team’s innovation objectives (Cardinal, 2001; Rickards et al., 2001; Gilson and Shalley, 2004). Second, distributive justice has an instrumental effect on the team. When the team holds the perception that rewards and resources are allocated fairly, it fosters certainty that hard work will realize rewards and resources in the future. Higher certainty enables team members to strive for excellence in their tasks (West, 2000). When the team strives for excellence in performing tasks aligned with a shared vision for innovation, the team members reflect on one another’s ideas by actively providing feedback for the successful implementation of ideas (Shalley, 2002; Hülsheger et al., 2009).

In contrast, distributive injustice evokes perceptions of inequity, which harms team members’ motivation to engage in innovation diverting attention to addressing perceived inequities (Simmons, 2011). The team perceives innovation goals as demanding and stressful (Janssen, 2004). Perceived outcome injustice harms intra-team synergy, lowers members’ willingness to share ideas, and reduces collaborative efforts in implementing ideas. Perceived injustice in the allocation of resources hinders team innovation due to a lack of access to necessary facilities, information, and funds, which are vital for creativity and innovation (Amabile, 1988).

*Proposition 1a: Within teams, state distributive justice climate level will positively relate to team innovation across time.*

#### State procedural justice climate level and team innovation

State procedural justice climate level refers to a *team’s in-the-moment or localized (episodic) shared perceptions (the mean) for fairness of organizational procedures and processes*. Individuals perceive procedural fairness as they evaluate whether organizational procedures and processes are consistent, accurate, unbiased, representative, ethical, and correctable (the six rules for procedural justice, Leventhal, 1980, Colquitt, 2001). Instead of focusing on instrumental aspects in distributive justice, procedural justice focuses on relational aspects (Lind and Tyler, 1988). Employees feel valued when they are allowed to contribute their voices to organizational procedures and processes (Tyler and Blader, 2005). Procedural justice climate fosters cooperation (Deery and Iverson, 2005), group identification (Lipponen and Wisse, 2010), positive job attitudes (e.g., pride, commitment, and satisfaction), and helping among team members (Hülsheger et al., 2009). In a supportive and cooperative work atmosphere, team members willingly help each other and collaborate in problem-solving, promoting team innovation (Amabile et al., 1996; Tiwana and McLean, 2005).

State procedural justice climate enables higher participative safety in a team. Participative safety indicates the extent to which members of a team are involved in decision-making and information/idea sharing (West and Anderson, 1996). In situations, where team members feel that they are encouraged to participate and freely speak up in decision-making, they are likely to invest higher effort in their work (West and Anderson, 1996). Participatory safe contexts build a non-threatening psychological atmosphere, in which team members feel trusted (Edmondson, 1999) and are more inclined to contribute new ideas since they need not worry about negative judgment by others (West, 1990). Furthermore, participatory safe contexts foster intra team activities pertaining to planning, implementation, and team learning such as sharing individual, mutually monitoring performance, communicating with peers, discussing errors openly, asking questions, and seeking feedback (Edmondson, 1999), which are likely to enhance team innovation.

*Proposition 1b: Within teams, state procedural justice climate level will positively relate to team innovation across time.*

#### State interactional (interpersonal and informational) justice climate level and team innovation

State interactional justice climate level refers to the *team’s in-the-moment or localized (episodic) shared perceptions (the mean) for fairness of respectful treatment, and truthful and adequate informational explanations for the decisions* (Bies and Moag, 1986; Greenberg, 1993). At the state level, shared perceptions of fair interpersonal treatment provide information that the group is valued and respected by the organizational authorities. Similar to procedural justice, interactional justice focuses on relational aspects. However, procedure justice signals that the team is valued by the organization—the system, whereas interactional justice signals that the team is valued by the authority figures—the actors (Bies and Moag, 1986; Fassina et al., 2008).

Respectful treatment received from the higher authority fosters team cohesiveness and members engage in positive interpersonal behaviors by cooperating and helping one another (Ehrhart and Naumann, 2004). Cohesion is said to be an important predictor of innovative work behavior in individuals (West and Farr, 1989; Woodman et al., 1993). Cohesiveness in the team instills feelings of belongingness and attachment among team members, facilitates the work environment, and supports members to interact with each other and exchange ideas (King et al., 1991; West and Wallace, 1991). Moreover, individuals tend to willingly take risks in situations where they perceive that they have reliable relationships and bonds with other members because they feel secure that they can rely on the team for support in exploratory and innovation-related activities (West, 1990).

Conversely, state interactional injustice climate level leads to increased conflicts and less cooperation (Tepper, 2000). Although task-related conflicts are found to positively associate with team innovation, relational conflicts in a team are detrimental to team innovation (Hülsheger et al., 2009). Since interactional justice is relational, low levels of collective interactional justice fuel relational conflicts among team members. Relational conflicts result in narrowing the range of attention, producing rigid thinking, and reducing cognitive complexity because members’ attention is diverted to relationships (Deutsch, 1969; Carnevale and Probst, 1998). By escalating relational displeasure, interactional injustice hinders communication, information processing, and team learning, which is detrimental to team innovation (Baron, 1991; Jehn, 1995; Pelled, 1996).

*Proposition 1c: Within teams, state interactional justice climate level will positively relate to the team’s innovative behaviors across time.*

### Justice climate and team innovation: Mediating role of state team affect

Team affect is defined as an affective experience that is shared, or held in common, by the members of a group or team (Barsade and Knight, 2015). Taking a bottom-up approach toward the formation of group affect, Barsade and Gibson (1998) suggested that group affect emerges when individuals share their affective expressions and experiences, which spread among all group members. Affect sharing in a team occurs through implicit and explicit emotion-sharing among team members (Kelly and Barsade, 2001). Implicit emotion-sharing occurs automatically without deliberate intent through subconscious processes including emotional contagion, vicarious affect, behavioral engrailment, and interactional synchrony (Kelly and Barsade, 2001). Emotional contagion indicates sharing and spreading of moods and emotions from one individual to another individual (Hatfield et al., 1994). Vicarious affect is experienced when emotions are aroused in observers by seeing the affective expressions of others (Bandura, 1986). Behavioral entrainment and interaction synchrony indicates the process whereby one individual adjusts or modifies his/her behavior unconsciously in order to coordinate or synchronize with other/s (Kelly, 1988). On the other hand, explicit emotion-sharing occurs as team members deliberately attempt to manipulate the emotions of other team members by intentional affective induction and affective impression management (Kelly and Barsade, 2001).

Justice events are affect-inducing (Cohen-Charash and Byrne, 2008; Colquitt et al., 2013). At the individual level, justice events trigger affective responses for several reasons. First, individuals form perceptions of justice or injustice as part of the appraisal process by evaluating situations as pleasant or threatening, which in turn leads to negative or positive emotions (Weiss et al., 1999; Cropanzano et al., 2000; Krehbiel and Cropanzano, 2000; Greenberg, 2004). Second, individuals evaluate justice-related events in terms of counterfactuals—would, could, and should—to answer whether the event could have been avoided, should have been avoided, and that an alternative would have yielded better outcomes (Folger and Cropanzano, 2001). These perceptions of unfairness lead to emotional reactions among individuals. Third, individuals also experience vicarious emotions when they witness unjust events (Folger and Skarlicki, 2008), At the team level, Degoey (2000) argued that one of the ways in which social contagion of justice was manifested through emotional contagion. Given that justice events are affect-inducing; the team members are likely to be influenced by one another’s emotional responses to justice-related events. The members of a group have a tendency to affiliate with other group members when faced with justice-related events in order to seek social validation for their emotional reactions. Accordingly, we posit the following,

*Proposition 2a: State justice climate level is positively related to state positive team affect level.*

*Proposition 2b: State injustice climate level is positively related to state negative team affect level.*

### Justice climate, positive team affect, and innovation: Role of playful interactions

In-the-moment or localized collective perceptions of justice induce positive emotions in the team at the collective level (Proposition 2a). Positive affect refers to a state of happiness, joy, enthusiasm, and pride (Watson and Tellegen, 1985; Larsen and Diener, 1992). Positive team emotions trigger social interactions that broaden and build the scope of attention and resources of the team (Fredrickson, 2001; Rhee, 2007). A positive team affective state broadens the range of attention, cognition, and action of team members, and builds the shared collective resources of the team (Rhee, 2007). Positive team affect enables playful interaction among members of a team (Fredrickson, 1998; Rhee, 2007). Such playful team-member interactions that are broadening and building comprise cognitive and social spontaneity among team members (Lieberman, 1977; Klein, 1980; Barnett, 1990). Cognitive spontaneity implies that team members engage in unconventional and imaginative ways of thinking. Members show curiosity and inventiveness while approaching problem-solving. The members are likely to *build on each other’s ideas* as their mindset is flexible and novelty-seeking (Singer et al., 1980; Rhee, 2007). Social spontaneity fosters relation-oriented interactions among members such as supportive *communication* and *affirmation* of others’ ideas (Rhee, 2007). Members use encouraging tones and gestures on one another’s ideas which foster team membership and a sense of collective identity (Jehn and Shah, 1997). Affirmation indicates that members approach others’ ideas and opinions in an encouraging and positive way (Rhee, 2007). Thus, the state-positive team affect enhances team innovation—idea generation by enabling amicable member interactions building on one another’s ideas, leveraging positive communication, and allowing affirmation of others’ ideas and opinions (Rhee, 2007). Accordingly, we posit the following,

*Proposition 3a: The positive effect of state justice climate level on team innovation—idea generation is serially mediated via (a) state positive team affect level and (b) intrateam playful interactions—information sharing, building on ideas, affirmation, and communication.*

### Justice climate, negative team affect, and innovation: Role of outcome-focused interactions

In-the-moment or localized collective perceptions of organizational injustice induce negative group emotions in the team (Proposition 2b). The research shows that owing to negativity bias, justice—adherence to fairness/rules, and injustice—violations to fairness/rules, trigger different responses among individuals (Colquitt et al., 2015). Negative stimuli are more salient, trigger more holistic assessments, and enable elaborated, descriptive and comprehensive analyses than positive stimuli (Baumeister et al., 2001). Individuals’ affective reactions and actions to injustice are different from their responses to justice (Colquitt et al., 2015). Whereas justice events, by large, induce pleasantness, injustice events trigger *contingent thinking* in addition to negative emotional responses such that individuals engage in situation-based or context-based evaluation of the event. Reactions to injustice are rich, detailed, and elaborated, including phases such as “it depends,” “either– or,” “if–then,” “sometimes this–sometimes that,” and “case by case” (Colquitt et al., 2015, p. 281).

The action tendencies arising from negative affect in response to state injustice climate level, thus, depend on the team’s blame attribution for the cause of injustice. People, in search of attributional information about who is responsible for the injustice incident, either internalize attribution by blaming the self or externalize attribution by blaming external entities. In this vein, people engage in counterfactual thinking in search of who is accountable for injustice, asking whether injustice incidents would, could, or should have been avoided (Folger and Cropanzano, 2001). Those individuals, who made external attributions, showed higher retaliation, resulting in outward-focused emotions (Barclay et al., 2005).

Internal blame attributions trigger the team members to experience inward-focused negative emotions (Barclay et al., 2005), which allows a narrowed scope of attention (Lazarus, 1991). In negative affective states, people restrict their attention and employ stringent categorization of tasks at hand. Rhee (2007) argues that negative team emotions are associated with outcome-focused interactions among team members leveraging on the narrowed scope of attention such that team members are likely to focus their attention on analyzing, critically evaluating, and completing the tasks at hand. Inward-focused negative emotions trigger action tendencies to turn unfavorable situations into favorable ones. Task-oriented or outcome-focused interactions—planning, monitoring, and critical evaluation emerge when team members narrow their focus of attention to the execution aspect of the tasks (Rhee, 2007).

Planning activities involve a specified and detailed breakdown of subtasks into procedures, the delegation of roles and responsibilities, time-based elaboration on the sequencing of task duties, and a detailed action plan (Weldon et al., 1991). Planning activities are aimed to define and explicate the actions to accomplish group tasks in time with efficiency. Monitoring activities involve checking the progress and quality of group performance (Jehn and Shah, 1997). Critical evaluation activities include “disagreements or arguments about the way a group member performs his/her duty, criticism about a member’s performance, or disapproval of a member’s suggestion” (Jehn and Shah, 1997, p. 778).

Planning, monitoring, and critical evaluation are relevant actions that positively associate with idea implementation. Since implementing new ideas carry ambiguity and uncertainty, team members use planning, monitoring, and critical evaluations in order to reduce risks in the future. Injustice brings negative emotions to team members. When members attribute the blame for such injustice internally, they try to correct the situation by engaging in planning, monitoring and critically evaluating the tasks at hand. Such action tendencies help team members to attain team innovation by fostering activities that enhance idea implementation. Accordingly, we postulate,

*Proposition 3b: The positive effect of state injustice climate level on team innovation—idea implementation is serially mediated via (a) state negative team affect level and (b) intrateam task-oriented interactions—planning, monitoring, and critical evaluation.*

### Justice climate, negative team affect, and innovation: Role of deviant interactions

External blame attributions trigger members to experience outward-focused negative emotions. Team members attribute the blame to the organization or the management. The outward-focused negative emotions trigger retaliatory tendencies among team members such that members try to engage in protests and deviant behaviors including silence, collective criticism, and group-based anti-social behaviors (Degoey, 2000).

#### Silence

Employee silence refers to the intentional withholding of critical work-related information by employees from their peers. At the collective level, team silence indicates that the members of a team intentionally withhold information, knowledge, and ideas from the other team members (Morrison and Milliken, 2000). In a broader sense, the employee chose to intentionally withhold their knowledge and avoid expressing their views, suggestions, and recommendations pertaining to organizational issues (Tangirala and Ramanujam, 2008). Research suggests that perceived organizational injustice promotes employee silence (Harlos, 2001; Pinder and Harlos, 2001; Tangirala and Ramanujam, 2008).

#### Collective criticism

Justice-rule violations often result in members engaging in collective rumination and group criticism, such as gossiping and spreading rumors (Bies, 2001; Folger and Skarlicki, 2008). Gossip is a collective form of criticism used by a group to discuss and make sense of norm- and justice- violations. One of the most commonly reported reactions among individuals who experience mistreatment is the desire and tendency to gossip (Degoey, 2000).

#### Group-based antisocial behaviors

Group-based antisocial behaviors are the team members’ attempts to “even the score” with the perpetrators—organization or organizational authorities who are attributed to the blame (Degoey, 2000). Such antisocial behaviors include theft (Greenberg and Scott, 1996), doing sloppy work, absenteeism (Lewicki et al., 1997), and direct/indirect revenge (Bies and Tripp, 1996). Unjust events trigger outward-focused team emotions such as anger, hostility, and frustration, which may bolster team members’ retaliatory tendencies (Skarlicki and Folger, 1997) against the perpetrator (or the entity of blame attribution) of the unjust treatment in a collaborative fashion.

When employees choose to remain silent, they withhold information from their team. Team innovation—idea generation and idea implementation require members to actively participate in team activities, and speak up about their ideas, concerns, and opinions. Thus, withholding information from the team shall negatively affect team innovation. Collective criticism is detrimental to team innovation since it diverts members’ attention to irrelevant activities, sapping resources—time and effort, which can be utilized in generating or implementing new ideas. Other antisocial behaviors including doing sloppy work, absenteeism, and purposive retaliation (Bies and Tripp, 1996; Greenberg and Scott, 1996; Lewicki et al., 1997) puts roadblocks to team innovation by adversely affecting the generation of new ideas and their adequate implementation. In all, we posit the following,

*Proposition 3c: The negative effect of state justice climate level on team innovation is serially mediated via (a) state negative team affect level and (b) intrateam counterproductive interactions—silence, collective criticism, and antisocial interactions.*

### Moderating role of state justice climate strength

Justice researchers argue that there is a distinction between justice climate level and justice climate strength (Naumann and Bennett, 2000; Colquitt et al., 2002). Whereas justice climate level denotes the extent to which members of the same unit believe that their unit as a whole has been treated fairly, justice climate strength depicts the degree of agreement among members of the same unit on whether the unit has been treated fairly (Li et al., 2015). Operationally, climate level is derived by aggregating scores of the justice perceptions from individual members within the same team where a higher average score depicts a more just climate. However, two teams with the same scores of justice climate level could have varying levels of agreement among the team members. For instance, one team report all members of moderate levels of justice perception/experience whereas the other may report some members of high justice perception/experience and others of low justice perception/experience, both the teams, however, will report similar justice climate level. Thus, justice climate strength refers to congruence in the team member’s perception of justice. At the state level, justice climate strength refers to low dispersion in team members’ momentary perceptions of organizational justice.^2^ Variability in intrateam justice perception could occur for a variety of reasons. First, not all individuals respond to (in) justice events in a similar manner. Individuals differ in personality dispositions including trust propensity, risk aversion, trait morality, and justice orientation (Colquitt et al., 2006), which influence how people perceive and react to (in) justice events. Second, the team members may experience distinct justice events in varying frequencies, which contributes to the variations in team members’ in-the-moment (or episodic) justice perceptions (Rupp and Paddock, 2010).

Operationally, a lower value of standard deviation (or the variance) of intrateam justice perceptions represents justice climate strength (Roberson, 2006). A few studies, however, devised more sophisticated analytic approaches such as dividing the standard deviation of team members’ justice perception by the mean level of the unit and then standardizing the ratio (Colquitt et al., 2002; Cropanzano et al., 2011) or calculating the interrater agreement index, r_wg_ (Naumann and Bennett, 2000). Whitman et al. (2012) meta-analysis further showed that justice climate strength moderates the relationship between justice climate level and team effectiveness such that low climate strength introduced ambiguity in how unit members interpret justice events and their implications, thereby lowering the effects of justice climate level on team effectiveness. Bigger effect sizes for the effect of justice climate level were found when justice climate strength was reported higher.

From this base, we argue that the relationship between the justice climate level and team innovation should be strengthened when justice climate strength is high. In a state of high congruence in justice perceptions, the higher justice climate level fosters team members to build on ideas, share ideas with one another and actively pursue their ideas for implementation. Similarly, the negative relationship between the injustice climate level and team innovation shall be strengthened when the injustice climate strength is high. In a state of high congruence in injustice perceptions, the higher injustice climate level instills more doubts among team members who are more likely to refrain from sharing ideas with one another and to actively promote their ideas for implementation, which in turn lowers team innovation. Accordingly, we posit,

*Proposition 4a: State justice climate strength moderates the relationship between state justice climate level and team innovation such that the positive relationship will be strengthened when state justice climate strength is high as compared to when it is low.*

*Proposition 4b: State injustice climate strength moderates the relationship between state injustice climate level and team innovation such that the negative relationship will be strengthened when state injustice climate strength is high as compared to when it is low.*

### Moderating role of state team affect diversity

Team affect diversity refers to the dispersion in the affective states of the team members. Dispersion in affective states occurs for various reasons. First, people differ in experiencing and expressing emotions based on their personality traits, including dispositional affect, proneness to empathy and expressivity, and the big 5 personality traits (Tiedens et al., 2004). Dispositional differences, such as trait negative affect and neuroticism can enhance tendencies to feel more negative affectivity; whereas trait positive affect and extraversion can promote tendencies to feel more positive affectivity in individuals. Thus, variations in team members’ affective responses occur due to individual differences. Second, variations occur due to the extent to which individuals identify with the team (Mackie et al., 2000). The members who strongly identify with the team are more likely to experience intense and vicarious emotions as compared to those who weakly identify with the team (Tiedens et al., 2004). From the earlier discussion in the previous section, we posit that different people should appraise justice-related events differently spurring dispersion in intrateam justice perceptions at the state level. Depending on how people appraise a justice event, they vary in emotional response to the justice event (Cohen et al., 1996). We argue that justice climate diversity should have a positive association with team affect diversity.

Justice events are affect-inducing (Cohen-Charash and Byrne, 2008; Colquitt et al., 2013). By emotional contagion, moods and emotions are shared and spread from one individual to other individuals (Hatfield et al., 1994) by vicarious affect when emotions are aroused in observers by seeing the affective expressions of others (Bandura, 1986). Social affiliation of emotions either results in polarization or mitigation of shared emotions. *Polarization* refers to the exacerbation of emotional reactions as a result of social facilitation and emotional tuning (Taylor et al., 1990). Individuals’ positive emotions will thus build into heightened collective positive team emotions. Similarly, individuals’ negative emotions will result in heightened collective negative team emotions. On the other hand, *mitigation* of emotions (e.g., negative emotions, anxiety) occurs when individuals find companionship by affiliating with other team members (Schachter, 1959). Companionship provides mutual comfort softening an individual’s emotional distress.

From this base, we argue that when state team affects diversity is low, the team members show a high similarity of congruence in their emotions or affective states. In such scenarios, the team member should tune into one another’s emotions quickly through emotional contagion triggering a *polarizing* effect that is the intrateam affect is exacerbated by emotional reactions from individual team members such that, collectively, the team members will report higher positive emotions or higher negative emotions (Taylor et al., 1990; Degoey, 2000). Team members’ positive emotional states associate positively with more amicable interactions among the members, whereas team members’ negative emotional states associate positively with counterproductive interactions among the members. We expect a bigger effect size for these relationships when state team affects diversity is low.

*Proposition 5a: State positive team affect diversity moderates the relationship between state positive team affect level and member’s playful interactions such that the positive relationship between state positive team affect level and member’s playful interactions will be stronger when state team affect diversity is low as compared to when it is high.*

*Proposition 5b: State negative team affect diversity moderates the relationship between state negative team affect level such that the positive relationship between state negative team affect level and member’s counterproductive interactions will be stronger when state team affect diversity is low.*

When state team affects diversity is high, team members diverge in their emotional experiences or state affectivity. In such scenarios, team-members tune into one another’s varying emotions inducing the *mitigation or normalization* effect. The mitigation or normalization effect is more pronounced in the context of negative emotions (e.g., anxiety, anger) such that social companionship lowers members’ levels of anxiety and anger as they affiliate with other team members (Schachter, 1959). From proposition 3b, we know that negative team affect positively associates with intrateam task-oriented interactions. We propose that diversity in the team affect is particularly relevant to this relationship. Team affect-diversity signals that the team members are likely to view the task/s from divergent perspectives. A perspective shapes how a situation (or a task or a problem) is viewed, to what extent it is perceived as relevant, and how the situation is evaluated from different aspects (Hoever et al., 2012). In a state where team members are likely to take divergent perspectives, the team can bring multiple solutions to resolve a problem, looking at it from different aspects. The differences in perspectives are a proximal indicator of the cognitive resources of a team (van Knippenberg et al., 2004). A team that holds a rich pool of diverse perspectives can look into the tasks in multiple ways. Diverse perspectives can equip a team with a wider set of approaches to the task (Jackson, 1992; Williams and O’Reilly, 1998). Furthermore, divergent perspectives can bring “disagreements among team members about the content of the tasks being performed, including differences in viewpoints, ideas, and opinions” (Jehn, 1995, p. 258). Such disagreements leverage information exchange, deliberation on opposing opinions, and critical evaluation of the tasks at hand (Brodbeck et al., 2002; West, 2002; Shalley and Gilson, 2004). Therefore, we posit,

*Proposition 5c: State negative team affect diversity moderates the relationship between state negative team affect level such that the positive relationship between state negative team affect level and intrateam task-oriented interactions will be stronger when state team affect diversity is high.*

Overview of the propositions is presented in Table 1.

**TABLE 1:** Overview of propositions.

	Proposition	Relationship
1a	Within teams, state distributive justice climate level will positively relate to team innovation across time.	State distributive justice climate level →Team innovation
1b	Within teams, state procedural justice climate level will positively relate to team innovation across time.	State procedural justice climate level →Team innovation
1c	Within teams, state interactional justice climate level will positively relate to team’s innovative behaviors across time.	State interactional justice climate level →Team innovation
2a	State justice climate level is positively related to state positive team affect level.	State justice climate level →Team affect level (PA)
2b	State injustice climate level is positively related to state negative team affect level.	State injustice climate level →Team affect level (NA)
3a	The positive effect of state justice climate level on team innovation—idea generation is serially mediated via (a) state positive team affect the level and (b) intrateam playful interactions—information sharing, building on ideas, affirmation, and communication.	State justice climate level →Team affect (PA) →Team interaction (Playful) →Team innovation
3b	The positive effect of state injustice climate level on team innovation—idea implementation is serially mediated via (a) state negative team affects the level and (b) intrateam task-oriented interactions—planning, monitoring, and critical evaluation.	State injustice climate level →Team affect (NA) →Team interaction (Task) →Team innovation
3c	The negative effect of state justice climate level on team innovation is serially mediated via (a) state negative team affects level and (b) intrateam counterproductive interactions—silence, collective criticism, and antisocial interactions.	State injustice climate level →Team affect (NA) →Team interaction (CWB) →Team innovation
4a	State justice climate strength moderates the relationship between state justice climate level and team innovation such that the positive relationship will be strengthened when state justice climate strength is high as compared to when it is low.	State justice climate level × State justice climate strength →Team innovation
4b	State injustice climate strength moderates the relationship between state injustice climate level and team innovation such that the negative relationship will be strengthened when state injustice climate strength is high as compared to when it is low.	State injustice climate level × State injustice climate strength →Team innovation
5a	State positive team affect diversity moderates the relationship between state positive team affect the level and member’s playful interactions such that the positive relationship between state positive team affect the level and member’s playful interactions will be stronger when state team affect diversity is low as compared to when it is high.	Team affect level (PA) × Team affect diversity →Team interaction (Playful)
5b	State negative team affect diversity moderates the relationship between state negative team affect level such that the positive relationship between state negative team affect level and member’s counterproductive interactions will be stronger when state team affect diversity is low.	Team affect level (NA) × Team affect diversity →Team interaction (CWB)
5c	State negative team affect diversity moderates the relationship between state negative team affect level such that the positive relationship between state negative team affect level and intrateam task-oriented interactions will be stronger when state team affect diversity is high.	Team affect level (NA) × Team affect diversity →Team interaction (Task)

## Discussion

### Theoretical implications

The proposed theoretical framework has several important implications for innovation literature. First, although scholars have proposed how teams within organizations can facilitate or inhibit innovation (Burningham and West, 1995; Drach-Zahavy and Somech, 2001; Hülsheger et al., 2009), almost all studies have examined the antecedents and the processes of team innovation taking a static view. Considering a static view of the team-level antecedents of team innovation is problematic because team innovation is, essentially, a dynamic process. For instance, at the organizational level, scholars have theorized and empirically examined the dynamics of organizational innovation in the two most studied models of innovation, the punctuated equilibrium (Gersick, 1991; Romanelli and Tushman, 1994) and ambidexterity (He and Wong, 2004; Andriopoulos and Lewis, 2009; Bledow et al., 2009) examining the dynamic interplay of organizational exploration and exploitation across time. Similarly, at the individual level, the dynamic perspective on the individual’s innovative behaviors across time has recently gained scholarly attention. Zacher and Wilden (2014) posit that ambidextrous leadership predicts employees’ daily self-reported innovative performance and successfully tested their predictions using a daily diary study. Bledow et al. (2013) showed an “affective shift” model of creativity postulating that, at the episodic level, an individual’s creativity is influenced by the dynamic interplay of positive and negative affect. Ng et al. (2010) found that innovation-related behaviors in employees were negatively affected by psychological contract breaches over time such that the increase in psychological contract breaches predicted a decrease in innovation-related behaviors. Given that innovation unfolds over time, scholars call for theoretical frameworks to explicate the dynamic processes underlying team innovation across time (Anderson et al., 2014). We advance the theory of innovation by explicating a dynamic processual model for team innovation.

Second, the effect of a justice climate on team innovation is examined in a very limited scope in the management literature. In particular, emphasis is given to procedural justice climate ignoring the possible influence of distributive and interactional justice climate on team innovation. Moreover, such effects are examined in the context of organizational change or change processes. The organizational changes are characterized as low-frequency high magnitude events. For example, the procedural justice climate (i.e., change process fairness) is found to foster a sense of control in the employees during organizational change (Caldwell et al., 2004), enhanced change commitment, and change self-efficacy (Herold et al., 2007) and positively associate with new product creativity and speed to market (Dayan and Colak, 2008). Although such research has advanced our knowledge considerably, it does not shed light on how “small” justice-related work events can influence team innovation. Broadening the research scope to discrete justice-related work events is important because team innovation is achieved in small steps by team members, who engage in innovation behaviors on a daily basis (Bledow et al., 2013; Zacher and Wilden, 2014).

The theoretical implications also expand to justice literature. Although justice scholars have elevated scholarship on how individuals form justice perceptions collectively and the consequences of justice climate at the unit level (Colquitt et al., 2005; Li and Cropanzano, 2009), one assumption is that once formed, the justice climate is stable, long-lasting, and relatively impervious to change. However, justice perceptions change on the daily basis (experience sampling and daily diary studies, Judge et al., 2006). In fact, it is argued that all daily events (e.g., cues and experiences), although at varying levels, provide information to individuals to build a general sense of fairness (Rupp and Paddock, 2010). Scholars notice that a limited focus is given to investigating the dynamic perspective at the team level (Fortin et al., 2016). We contribute to the emergent theories of justice, which are based on event-based justice formation and change across time.

Finally, only a few theoretical frameworks consider constructs of justice climate strength and team affect diversity in the team processes and outcomes. Variance in justice climate is predominantly studied for its properties associated with the lack of divergence in members’ justice perceptions and is termed as justice climate strength. Research shows that congruence in justice perceptions, over time, strengthens the relationship between justice climate level and team performance as the team develops shared norms (Li et al., 2015). Similar to justice literature, group affect literature also showed that trait affective diversity resulted in increased conflicts and decreased cooperativeness among top management teams (Barsade et al., 2000). An underlying assumption here is that, over time, teams become homogeneous. As we have pointed out earlier, such arguments build on the static view conforming to the trait affect or a stable justice climate. At the state level, however, diversity in group member’s justice perceptions and affective responses could have a significant impact as pointed out by a few researchers (Tiedens et al., 2004; Rupp and Paddock, 2010; Jones and Skarlicki, 2013; Fortin et al., 2016). We, therefore, contribute to the team affect and justice climate literature by elaborating how team affects diversity and justice climate strength influence team-level outcomes, in particular, intra-team interactions and team innovation.

### Limitations and future directions

The proposed theoretical framework is not without limitations. These limitations should be addressed in future theory development. First, justice scholars acknowledge that justice-related events have an accumulative effect such that people do not change their entity-based justice perceptions based on every event, instead, they notice the justice events over a period of time before reflecting changes in the justice perceptions for entities (Rupp and Paddock, 2010, Fortin et al., 2016). Therefore, there could be a feedback loop that prior justice-related events can influence concurrent justice perceptions. On a similar note, team affect scholars posit that momentary emotional experiences accumulate into forming history, which in turn affects how members appraise events and experiences in the future (Hareli and Rafaeli, 2008; Walter and Bruch, 2008). Therefore, future research can look into the historical effects of justice-related events and team affective states, and how they can influence team interactions and team innovation.

Second, organizational structure plays a significant role in predicting employees’ justice perceptions (Cropanzano and Greenberg, 1997; Schminke et al., 2000, 2002). For instance, the centralized and mechanistic organizational structure is perceived to be low in organizational justice (Schminke et al., 2000, 2002). In contrast, organic organizations foster trust via interactional justice. Since organic organizations are more decentralized, loose, and flexible in structure, they provide a conducive environment for innovation. The justice-innovation relationship in mechanistic organizations is, however, particularly important since there is a heightened interest among CEOs and top management teams to lead the organization into innovation *via* intrapreneurship. Therefore, future studies shall focus on explicating the dynamic justice-innovation relationship in the context of mechanistic and organic organizations.

Third, future research should elaborate on the interplay of organizational justice dimensions in predicted affective responses and, thus, team interactions and team innovation. Appraisal theory (Lazarus, 1991) contends that individuals appraise events in two stages, a generalized primary appraisal of whether an event is relevant or is positive/negative, and a detailed secondary appraisal analyzing whether an event could be avoided, who is responsible and how to cope with the event. This suggests a two-stage model of fairness (Weiss et al., 1999), which proposes that outcome favorability serves as a primary appraisal and procedural/interactional justice serves as the secondary appraisal. Several empirical research provides evidence that distributive justice, or outcome favorability, interacts with procedural and interactional justice to predict how employees will respond to organizational justice (Cohen-Charash and Byrne, 2008). Therefore, scholarship building upon the interplay of organizational justice dimensions will greatly contribute to the literature by shedding light on the complexities involved in the intersection of distribution, procedural and interactional justice in predicted team affective responses and, thus, team interactions and team innovation.

## Author contributions

NT conceptualized and developed the theory. SS significantly contributed to theoretical development and revision of the model. Both authors contributed to the article and approved the submitted version.

## Footnotes

1 Team or work-group refers to the purposive group defined as “an intact social system, complete with boundaries, interdependence for some shared purpose, and differentiated member roles” (Hackman and Katz, 2010, p. 1210).

2 Unless specified, in the rest of the article, “justice” or “organizational justice” refers to all three dimensions of the justice construct—distributive, procedural, and interactional justice.
